# Profiling of Plasma Extracellular Vesicle Transcriptome Reveals That circRNAs Are Prevalent and Differ between Multiple Sclerosis Patients and Healthy Controls

**DOI:** 10.3390/biomedicines9121850

**Published:** 2021-12-07

**Authors:** Leire Iparraguirre, Ainhoa Alberro, Thomas B. Hansen, Tamara Castillo-Triviño, Maider Muñoz-Culla, David Otaegui

**Affiliations:** 1 Multiple Sclerosis Unit, Biodonostia Health Research Institute, 20014 San Sebastián, Spain; leire.iparraguirre@biodonostia.org (L.I.); ainhoa.alberro@biodonostia.org (A.A.); TAMARA.CASTILLOTRIVINO@osakidetza.eus (T.C.-T.); 2 Research Institute for Medicines (iMed.ULisboa), Faculty of Pharmacy, Universidade de Lisboa, 1649-003 Lisboa, Portugal; 3 Molecular Biology and Genetics Department, Aarhus University, 8000 Aarhus C, Denmark; tbh@mbg.au.dk; 4 Spanish Network of Multiple Sclerosis, 08028 Barcelona, Spain; 5 Neurology Department, Donostia University Hospital, 20014 San Sebastián, Spain; 6 Department of Basic Psychological Processes and Their Development, University of the Basque Country, 20018 San Sebastián, Spain

**Keywords:** extracellular vesicles, plasma, transcriptome, RNA-Seq, circular RNAs, circRNAs, multiple sclerosis

## Abstract

(1) Background: Extracellular vesicles (EVs) are released by most cell types and are implicated in several biological and pathological processes, including multiple sclerosis (MS). Differences in the number and cargo of plasma-derived EVs have been described in MS. In this work, we have characterised the EV RNA cargo of MS patients, with particular attention to circular RNAs (circRNAs), which have attracted increasing attention for their roles in physiology and disease and their biomarker potential. (2) Methods: Plasma-derived EVs were isolated by differential centrifugation (20 patients, 8 controls), and RNA-Sequencing was used to identify differentially expressed linear and circRNAs. (3) Results: We found differences in the RNA type distribution, circRNAs being enriched in EVs vs. leucocytes. We found a number of (corrected *p*-value < 0.05) circRNA significantly DE between the groups. Nevertheless, highly structured circRNAs are preferentially retained in leukocytes. Differential expression analysis reports significant differences in circRNA and linear RNA expression between MS patients and controls, as well as between different MS types. (4) Conclusions: Plasma derived EV RNA cargo is not a representation of leukocytes’ cytoplasm but a message worth studying. Moreover, our results reveal the interest of circRNAs as part of this message, highlighting the importance of further understanding RNA regulation in MS.

## 1. Introduction

Extracellular vesicles (EVs) are membrane-coated particles of endosomal or plasma membrane origin that are secreted to the extracellular environment. Almost all cell types release EVs, both in physiological and pathological conditions, and they can be isolated from plasma and other bodily fluids. They play an essential role in indirect cell-to-cell communication, as their proteins, lipids and nucleic acids can be transferred between cells [1]. This EV-mediated communication has been shown to be implicated in the regulation of a number of biological functions, including immune response [2,3]. Moreover, they have been related to inflammatory and autoimmune diseases, including multiple sclerosis (MS) [2,4].

MS is a chronic autoimmune disease of the central nervous system (CNS) leading to demyelination. Consequently, it is associated with axonal and neuronal degeneration, resulting in neurological disability. It affects more than 2.5 million people worldwide and it is mainly diagnosed in young adults between 20 and 40 years [5]. MS is three times more prevalent in women than men, and there is evidence that this ratio may be increasing [6,7], a phenomenon shared with several other autoimmune diseases.

Different clinical forms of the disease are distinguished. For most of the patients (about 85%), the early phase of the disease is relapsing–remitting MS or RR-MS. RR-MS is characterized by clinical exacerbations, or relapses, caused by autoreactive immune cells that enter the CNS, resulting in focal inflammation and demyelination that produces neurological symptoms. After these relapses, which last at least 24 h, the inflammation resolves and partial remyelination occurs, entering a phase named remission. With time, for many RR-MS patients, the recovery starts to be incomplete, leading to disability accumulation. This later phase of the disease is characterized by less relapses but a progressive accumulation of disability, and is named secondary progressive MS (SP-MS). For the other 10–15% of the patients, the disease progresses from the onset with no apparent relapses, a course known as primary progressive or PP-MS [8,9,10].

Studies on EVs in MS patients have revealed a general change in their number and cargo when compared to controls, but also in relapse and in response to treatments [11,12,13]. In this context, EVs and their cargo have gained attention as potential biomarkers in MS in recent years.

Several components of the RNA cargo have been studied in different diseases including MS, revealing their importance in diagnosis [14] as well as for the assessment of disease activity [15], prognosis and treatment response [16]. It is worth noting that most of the studies have highlighted the relevance of noncoding RNAs (ncRNAs), and particularly microRNAs (miRNAs). Nevertheless, the EV RNA cargo is highly complex, with representation of most of the transcript types, and very few studies have addressed the full transcriptome content [17]. So far, only one study has characterized the whole EV transcriptome in MS [12]. However, recently, researchers have discovered that circular RNAs (circRNAs), an emerging family of endogenous ncRNAs, are enriched in EVs [18,19], and they have never been studied as part of the EV RNA cargo in MS. 

CircRNAs are formed by an alternative splicing process called backsplicing, by which a downstream splice donor and upstream splice acceptor are joined together. The resulting covalently closed circular transcripts are characterized by the lack of free ends and the presence of a circRNA-exclusive junction called back-splice junction (BSJ). Their circular structure endows them with their high stability, which along with their presence in different biofluids, their cell- or tissue-specific expression and their evolutionary conservation, has attracted the interest of many researchers [20,21]. These circRNAs have been reported to take part in a variety of physiological and pathological processes, and thus are part of the EV RNA cargo that is worth studying.

In fact, circRNAs have been detected in EVs of bodily fluids from different pathological conditions, and have been found to play a role in processes such as drug resistance, metastasis or cell proliferation [22]. Most studies focus on circRNAs in EVs in cancer [22,23], and thus insights into their role in other diseases are limited. Moreover, in most cases, the molecular function exerted by circRNAs in the pathological process remains unknown. For some other cases, the sponging of specific miRNAs [24,25], the sequestering or interaction with particular proteins [26,27,28], or the peptide synthesised by a circRNA [29,30,31] have been suggested as causative factors of different diseases such as neurologic, cardiovascular, immune diseases or cancers [32,33,34,35,36,37].

In this work, we have characterized the transcriptomic profile of EVs in MS patients and healthy controls, including for the first time the characterization of circRNAs and a preliminary study of their potential functions in the disease. Moreover, we have compared some of the features of the EV transcriptome with the transcriptome in leukocytes from MS patients and healthy controls recently described by our group [38].

## 2. Materials and Methods

### 2.1. Blood Sampling

For the study of EV transcriptome, whole blood was obtained from a total of 10 MS patients classified as RR-MS patients, 10 SP-MS patients, and eight sex- and age-matched healthy controls (HC) in the Department of Neurology at the Donostia University Hospital. All donors provided written informed consent prior to sample extraction. The main clinical and demographical characteristics of both patients and healthy donors are summarised in Table 1. The study was approved by the hospital’s ethics committee (UEM-IMN-2017-01) and part of the samples have been processed and stored at the Basque Biobank (www.biobancovasco.org, last accessed on 1 March 2019).

Peripheral blood was collected by venipuncture in EDTA tubes (Vacutainer, BD Biosciences) and centrifuged at 1258× *g* for 20 min to separate the plasma from the cellular fraction. Plasma was carefully collected, aliquoted and stored at −80 °C.

### 2.2. EV Isolation and RNA Extraction

EVs were isolated following a differential centrifugation step protocol as previously described by our group [39]. Briefly, plasma aliquots were centrifuged at 13,000× *g* for 2 min, supernatant was transferred to a new tube and centrifuged again at 20,000× *g* for 20 min to pellet EVs. The pellet was then resuspended with 100 µL of DPBS (GIBCO, ThermoFisher, Waltham, MA, USA). The DPBS had previously been double-filtered through a 0.22 µm-pore filter in order to remove particles and aggregates. RNA was directly isolated from the EV samples by using Trizol LS (ThermoFisher, Waltham, MA, USA) following the manufacturer’s protocol. RNA was quantified by NanoDrop ND-1000 spectrophotometer (ThermoFisher, Waltham, MA, USA).

### 2.3. Nanoparticle Tracking Analysis (NTA)

The size distribution and concentration of isolated plasma EVs were measured using a NanoSight LM10 device (Malvern Panalytical, Malvern, UK), as described elsewhere [40]. Samples were diluted to appropriated levels to obtain accurate acquisitions. Camera settings were fixed and maintained for all samples. Filtered DPBS was tested, and no background signal was detected. For each sample, two videos of 1 min were recorded and analysed with NanoSight NTA software 2.2 (Malvern Panalytical). 

### 2.4. Cryoelectron Microscopy (Cryo-EM)

For visualization of the isolated EVs, samples were vitrified following standard protocols [41]. Glow-discharged Quantifoil holey carbon film grids (Orthogonal Array of 2 μm Diameter Holes—2 μm Separation, mounted on a 300 M Cu grid, #657-300-CU, Ted Pella) were vitrified in liquid ethane in Vitrobot (FEI) after deposition of 3 μL of sample. Cryotransfer sample holders of the type GATAN Model 626 were used to keep the sample vitrified during electron microscopy analysis. The sample was observed in a JEM-2100F UHR (80–200 kV, JEOL, Ltd., Tokio, Japan) field emission gun (FEG) transmission electron microscope at different magnifications. Micrographs were recorded on TVIPS F216 CMOS camera (2 k × 2 k).

### 2.5. RNA-Seq

For library preparation and sequencing, a minimum input of 2 µg RNA was required. In order to fulfil this criterium, RNA concentration was measured by Nanodrop ND-1000 spectrophotometer (ThermoFisher, Waltham, MA, USA) and samples were pooled in pairs or trios of samples with the same disease status and sex. A total of 12 pools were obtained, 4 RR-MS pools, 4 SP-MS pools and 4 HC pools. 

The concentration and quality of the RNA pools was measured using Bioanalyzer 2100 instrument (Agilent Technologies, Santa Clara, CA, USA) before library preparation at CD Genomics (Shirley, New York, NY, USA). After normalization, rRNA was depleted from the total RNA sample using the Ribo-Zero rRNA removal kit (Illumina, San Diego, CA, USA) and followed by purification and fragmentation steps. To construct the sequencing libraries, a strand-specific cDNA synthesis was performed, the 3′ ends were adenylated, and adaptors were ligated. The resulting libraries were subjected to standard quality control and normalization processes. Paired-end sequencing was performed with Illumina HiSeq X Ten (Illumina, San Diego, CA, USA), and an average of 40–50 × 10^6^ reads were obtained per sample. All the steps explained above are schematically shown in Figure S1, from sample preparation to detection and differential expression explained in the following sections.

### 2.6. CircRNA Detection and Quantification in RNA-Seq Data

Sequencing reads were quality checked and mapped to the hg19 using BWA [42] or Bowtie2 [43] for subsequent analysis with CIRI2 and find_circ, respectively. Subsequently, circRNA prediction was performed by find_circ, version 1.0 [44], and CIRI2 [45], adhering to the recommendation by the authors. For find_circ [44], an increased stringency threshold was used, requiring that both adaptor sequences map with the highest possible mapping quality (mapq = 40). Moreover, only circRNAs supported by at least two reads in a given pool and found by both algorithms were used in subsequent analyses. CircRNA expression was based on back-splice junction (BSJ)-spanning reads according to CIRI2 quantification. Differential expression analysis was performed using DESeq2 [46] in R-studio, specifying a design formula (~disease status + sex) that controls the effect of the sex while comparing the different conditions (RR-MS vs. HC, SP-MS vs. HC or SP-MS vs. RR-MS).

CircRNAs detected in at least one pool for each of the groups compared, with an absolute fold-change (FC) value higher than 1.5 (FC > |1.5|) and a *p*-value less than 0.05 (*p* < 0.05) were considered differentially expressed circRNAs (from now on, DE circRNAs). CircRNAs detected in at least one pool for a given group and absent in all the pools comprising the other group included in the comparison were considered as group-exclusive circRNAs.

### 2.7. Linear Transcript Detection and Quantification in RNA-Seq Data

After the quality check, the sequencing reads were mapped to the hg19 with STAR [47] using default parameters. HTSeq [48] was used to quantify expression of gencode annotated genes (gencode v28) in a strand-specific manner. Read counts were then used for gene-level differential expression analyses using DESeq2 in R-studio, and as defined for circRNAs, differentially expressed linear RNAs (DE linear RNAs) were defined as those detected in at least one pool for each of the groups compared, with a FC > |1.5| and *p*-value < 0.05. Linear RNAs detected in at least one pool for a given group and absent in all the pools comprising the other group included in the comparison were considered as group-exclusive transcripts.

### 2.8. Classification of Transcript Types

CircRNAs were defined as those detected with at least two reads by CIRI2 and find_circ. For linear transcripts, gene type information was obtained from the Biomart database. For transcripts that were not classified in Biomart, gene type information was manually completed based on GeneCards, Ensembl and Lncipedia. Twelve transcript type categories were finally defined: protein coding transcript, long noncoding RNA (lncRNA), pre-microRNA (pre-miRNA), miscellaneous RNA (such as RNAs involved in important ribonucleoprotein complexes implicated in the transcription and translation processes), small nuclear RNA (snRNA), small nucleolar RNA (snoRNA), small Cajal body-specific RNA (scaRNA), ribosomic RNA (rRNA), transfer RNA (tRNA), circRNA, other RNAs (including pseudogenes, ribozymes, mitochondrial RNAs, etc.) and nondefined RNAs.

For each transcript type, the mean number of reads was calculated for each condition (RR-MS, SP-MS and HC) by calculating the mean for the raw read count of the samples comprising each group. Statistical difference between distributions was calculated by chi-squared test, performed using R studio.

### 2.9. Identifying circRNAs with Potential to Be miRNA Sponges

Dudekula et al. defined circRNAs with more than 20 miRNA binding sites (BS) as “super-sponges” for miRNAs [49]. Thus, to determine the potential of circRNAs to be miRNA sponges, we retrieved from CircInteractome [49] the miRNA–circRNA interactions for circRNAs with more than 20 miRNA binding sites. CircInteractome employs the TargetScan algorithm [50] to predict miRNAs that target circRNAs by surveying for 7-mer or 8-mer complementarity to the seed region, as well as the 3′ end of each miRNA. Based on this dataset, we assessed the number of miRNA binding sites for all the circRNAs detected by intersecting the datasets using R in RStudio. To evaluate whether the observed distribution of miRNA binding sites and the expected one were different, we used a chi-squared test, performed using R in RStudio.

### 2.10. CircRNA Structure Determination

The sequence of an RNA molecule is usually not sufficient for a reliable structure prediction. However, its combination with experimental structure data obtained from methods based on the study of structure-specific chemical modifications such as the SHAPE-MaP [51] method used by Liu et al. [28], or DMS-MaP [52] used by Fischer et al. [53], can result in an accurate assessment of the RNA folding status and RNA structure. Fischer et al. have recently found that the in silico-calculated length-normalized minimum thermodynamic free energy (−∆G/nt) of an RNA molecule has a very good correlation with the structures determined by DMS-Map, therefore, we followed the same methodology for the structure determination of circRNAs [53].

The CircRNA sequences was obtained from CircInteractome. The sequence was folded in silico using the RNAfold function from Vienna package 2.0. to calculate the minimum thermodynamic free energy, and later normalized with the spliced length of the circRNA to calculate the −∆G/nt value. Based on this value, circRNAs were classified as highly structured (−∆G/nt > 0.25) or poorly structured (−∆G/nt < 0.2). Statistical difference between distributions was calculated by chi-squared test, performed using R studio.

### 2.11. Gene Ontology Analysis

Gene ontology analysis has been performed by ShinyGO v0.741 (Brookings, SD, USA) [54] using the DE genes (*p*-value < 0.05 and FC > |1.5|.

## 3. Results

### 3.1. Characterization of Plasma Isolated EVs

**Figure 1:**
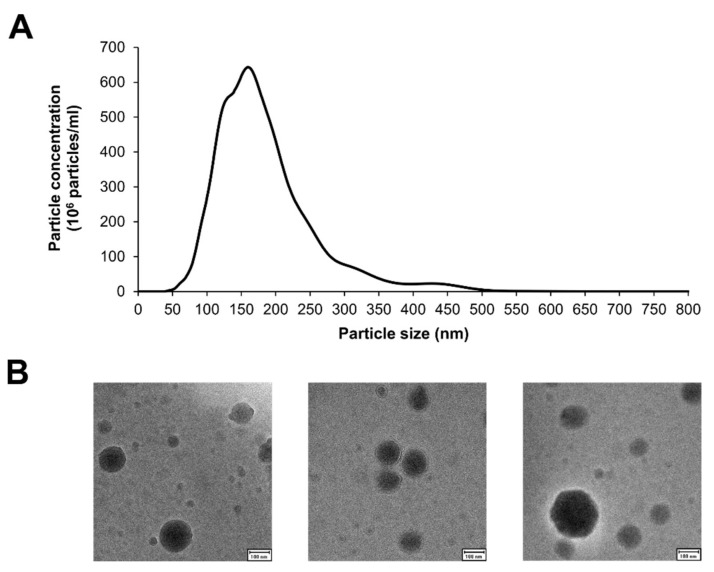
Plasma-derived EV characterization. (**A**) Representative figure of particle length distribution of EVs obtained by NTA. (**B**) Representative cryoEM images of EVs isolated following a differential centrifugation step protocol. Scale bar: 100 µm.

NTA results show a particle size distribution that confirms efficient isolation of small EVs and removal of larger particles such as plasma platelets, with most vesicles ranging between 50 and 300 nm (Figure 1A). Moreover, the presence of EVs and their mean size, as well as their rounded shape could be confirmed by cryo-EM (Figure 1B).

### 3.2. Both Linear and Circular Transcripts Are Abundantly Detected in Plasma-Derived EVs

For the RNA profiling of EVs from MS patients and HC, we performed an RNA-seq of rRNA-depleted total RNA from plasma-derived EVs of 20 MS patients and eight HCs divided into four RR-MS pools, four SP-MS pools and four HC pools. Find_circ and CIRI2 detected a total of 10,906 and 17,542 unique circRNAs supported by at least two BSJ spanning reads, respectively. A good detection overlap was observed between both algorithms, with 6575 circRNAs in common, which were classified as bona fide circRNAs. The linear transcriptome profile of EVs was also studied, and linear RNAs from 18,372 different genes were detected using HTSeq.

**Figure 2:**
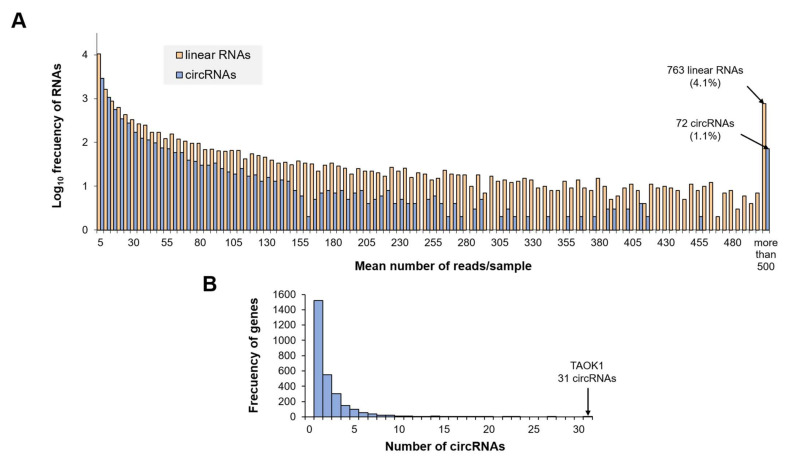
General features of the transcriptome profile in Evs. (**A**) Histogram showing the number of reads per circular or linear transcript. For clarity, the RNA frequency has been log scaled (y axis), and for those transcripts with a mean of more than 500 reads/sample the accumulated frequency is depicted. (**B**) Histogram showing the number of circRNAs produced from each gene. This section may be divided by subheadings. It should provide a concise and precise description of the experimental results, their interpretation, as well as the experimental conclusions that can be drawn.

Most of the circular and linear transcripts were modestly expressed, with 60.7% of the circRNAs and 65.6% of the linear RNAs detected with a mean of less than 10 reads per sample. Among the rest of the transcripts with a Base Mean ≥ 10 reads/sample, it is worth noting that 3214 linear transcripts (17.5%) were detected with a mean of more than 50 reads/sample, out of which 763 (4.1%) are highly abundant transcripts, accounting for more than 500 reads/sample. Among the circRNAs, highly abundant circRNAs have also been detected, although their proportion is smaller. A total of 819 circRNAs (12.5%) were detected, with an average of more than 50 reads per sample, and among those was a small group of 72 circRNAs (1.1%) that stands out as highly abundant (Base Mean ≥ 500 reads/sample) (Figure 2A).

Nevertheless, it is worth noting that linear transcripts were quantified at gene level while circRNAs were not. Out of the 6575 bona fide circRNAs detected, 206 circRNAs are intergenic and have not been assigned to any host-gene, and the remaining 6369 are located in a known gene locus. However, only 2814 different host-genes were identified, revealing that some genes produce more than one circular transcript. In fact, 54.1% of those genes give raise a single detectable circRNA transcript, while 44.3% produce between 2 and 10 circRNAs. There is a small subset of genes (1.5%) from which more than 10 different circRNAs are generated and incorporated into EVs, with a maximum of 31 circRNAs produced from the gene coding for the serine/threonine-protein kinase TAO1 (TAOK1) (Figure 2B).

### 3.3. CircRNAs Are the Second Most Abundant RNA Transcript in EVs, However Not in Leukocytes

**Figure 3:**
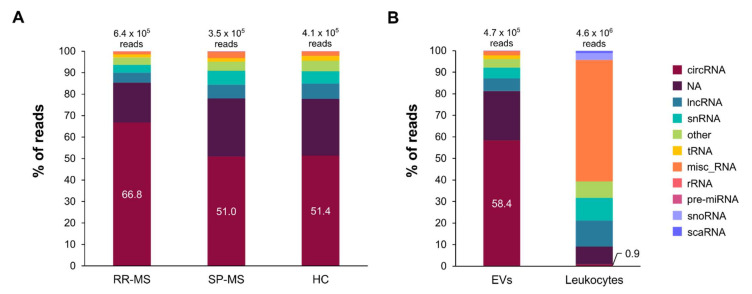
Distribution of ncRNA types in the EV transcriptome profile of MS patients and controls. In order to improve the visualization of the rest of RNA types, protein-coding transcripts which represent the highest proportion have been omitted from the graph. The proportion of the different ncRNA transcript types are represented by their corresponding % out of the total ncRNA reads. The mean number of ncRNA reads for each of the conditions are shown on top of the bars. The % of circRNAs is also depicted in the corresponding portion of the bar. (**A**) Distribution of the ncRNA types in EVs from RR-MS, SP-MS and HCs. (**B**) General distribution in EVs and leukocytes. A significant difference is found for the distribution of circRNAs and miscellaneous RNAs between EVs and leukocytes (*p* < 0.0001).

Taking together all the linear and circular transcripts, we have analysed more than seven million reads over 15,000 different transcripts per sample pool. In order to characterize the complete transcriptome in EVs, we categorized those transcripts into 12 different types, as defined in the materials and methods section. All 12 transcript types were present in all the samples, but different distributions have been observed. Obtained data show that protein-coding transcripts are the most prevalent in plasma-derived EVs, accounting for more than 92.9% of the total reads per sample. Interestingly, among the rest of non-protein coding RNAs (Figure 3), circRNAs are by far the most frequent, representing more than half of this group. This distribution does not differ between the RR-MS, SP-MS and HC groups, although circRNAs are slightly more frequent in RR-MS patients (66.8% compared to 51% and 51.4%) (Figure 3A). Among the rest of the ncRNAs that could be categorized, lncRNAs are the second most frequent, accounting for 4.7–7.1% of the ncRNAs, whereas pre-miRNAs, snoRNAs, and scaRNAs, among others, represent less than 1%, respectively. It is worth noting that although rRNAs are detected, their frequency is also less than 1%, confirming that although the rRNA depletion performed in the samples was not 100% efficient, they were almost completely removed.

First, the mean number of reads in leukocytes is 19.7 × 106 reads, higher than in EVs with a mean of 6.6 × 106 per sample (almost three times higher). In both cases, protein-coding transcripts are the most prevalent, but they represent a smaller proportion in leukocytes (76.6%, compared to 92.9% in EVs). Regarding the ncRNA part, in contrast with what we found for EVs, circRNAs only account for 0.9% of the reads (compared with 58.4% in EVs) (*p* < 0.0001). In leukocytes, miscellaneous RNAs are the ones that are more abundant among the non-protein-coding transcripts (56.2%), a surprisingly high proportion that is not reflected in EVs, where they only account for 1.9% of the reads (*p* < 0.0001) (Figure 3B).

### 3.4. The circRNA Profile in EVs from MS and Controls Is Different, as Is the circRNA Profile from RR-MS and SP-MS Patients

With the hypothesis that patients diagnosed with RR-MS and SP-MS could present a different EV transcriptomic profile, we performed three different comparisons: RR-MS vs. HC, SP-MS vs. HC and SP-MS vs. RR-MS.

**Figure 4:**
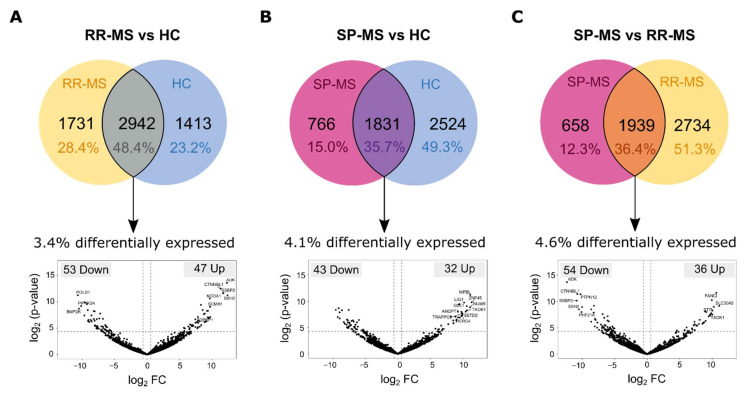
Differentially expressed circRNA. Venn diagrams showing the number of specific and common circRNAs detected in groups RR mm and HC (**A**), SP-MS and HC (**B**), and SP-MS and RR-MS (**C**), respectively. The percentage of group-exclusive and common circRNAs is calculated out of the total number of circRNAs for each comparison and depicted in the corresponding section of the Venn diagram. Below, volcano plots showing the log2-fold change in expression and associated *p*-values between groups as denoted. The number of significantly upregulated and downregulated circRNAs (FC > |1.5|; *p*-value < 0.05) are also shown in the upper right and upper left corners for the volcano plots, respectively.

When we compared the circRNA profile between RR-MS patients and HC, we found that among the 6575 bona fide circRNAs detected in total, 1731 (28.4%) were exclusively detected in RR-MS samples, whereas 1413 circRNAs (23.2%) were exclusive to HCs (Figure 4A). Further, from the 2942 circRNAs that have been detected in RR-MS and HC simultaneously, 100 circRNAs were found to be differentially expressed (FC > |1.5| and *p*-value < 0.05), with 47 of them upregulated (seven upregulated circRNAs with *p*-adj < 0.05) and 53 downregulated in RR-MS patients (four downregulated circRNAs with *p*-adj < 0.05) (Figure 4A).

Regarding the comparison between SP-MS patients and HC, it is worth noting that due to the small number of circRNAs detected in SP-MS patients, the biggest proportion of circRNAs, accounting for 49.3% (2524 circRNAs), were only detected in HCs, while the SP-MS-specific profile included 766 circRNAs (15%) (Figure 4B). From the remaining 35.7% of circRNAs found in common between both groups, 32 circRNAs were found to be upregulated (seven upregulated circRNAs with *p*-adj < 0.05) in SP-MS patients and 43 downregulated (one downregulated circRNAs with *p*-adj < 0.05) (Figure 4B).

Results drawn for these two comparisons indicate that there is a different profile of circRNAs in plasma-derived EVs from MS patients when compared to controls. However, in order to assess whether EV transcriptome profiles from SP-MS and RR-MS patients are also different, we also compared these two groups. We found that there are 1939 circRNAs (36.4%) in common between both MS types, although the expression of some of them is changed between both group. In fact, 36 circRNAs were found to be significantly upregulated in SP-MS patients (four upregulated circRNAs with *p*-adj < 0.05), and 54 downregulated (six downregulated circRNAs with *p*-adj < 0.05) (Figure 4C). Moreover, 2734 circRNAs were exclusive to those diagnosed with RR-MS, and 658 circRNAs were only found in SP-MS patients (Figure 4C). Table 2 shows a list of potential candidates. The best five transcripts expressed in both groups, selected by the *p*-corrected value, and the candidates most expressed in one group and absent in the other, have been selected.

### 3.5. The Linear Transcriptome in EVs Differs between Different MS Types and Controls

In the same way that the circular transcriptome is different when we compared EVs from RR-MS, SP-MS and HC individuals, the linear transcriptome was also found to be different.

**Figure 5:**
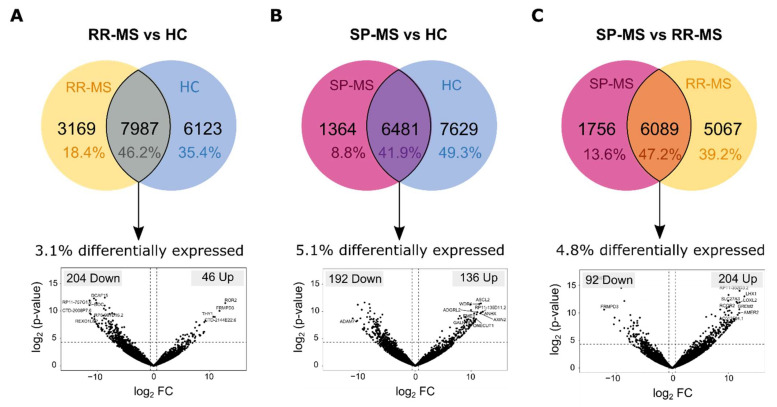
Differentially expressed linear RNA. Venn diagrams showing the number of specific and common linear RNAs detected in groups RR-MC and HC (**A**), SP-MS and RR-MS (**B**), and SP-MS and RR-MS (**C**), respectively. The percentage of group-exclusive and common linear RNAs is calculated out of the total number of linear RNAs for each comparison and depicted in the corresponding section of the Venn diagram. Below, volcano plots showing the log2-fold change in expression and associated *p*-values between groups as denoted. The number of significantly upregulated and downregulated linear RNAs (FC > |1.5|; *p*-value < 0.05) are also shown in the upper right and upper left corners for the volcano plots, respectively.

The comparison between RR-MS patients’ and HCs’ linear transcripts reveals that there are nearly twice as many HC-exclusive linear RNAs (6123) as there are in the RR-MS group (3169) (Figure 5A). Additionally, in line with this result, most of the common linear RNAs found to be differentially expressed were downregulated (204 linear RNAs, 81.6%) (16 downregulated linear RNAs with *p*-adj < 0.05), and only 46 were found to be upregulated in RR-MS patients (two upregulated linear RNAs with *p*-adj < 0.05) (Figure 5A).

Regarding the case of SP-MS patients, similarly to what was observed for circRNAs, the number of linear RNAs detected in SP-MS patients is smaller than the number detected in the rest of the groups. Consequently, the number of group-specific linear transcripts is also smaller when compared to HCs (1364 transcripts (8.8%) in SP-MS vs. 7629 transcripts (49.3%) in HCs). For the linear RNAs detected in both groups (6481 transcripts), 136 transcripts are found to be upregulated (22 upregulated linear RNAs with *p*-adj < 0.05) in SP-MS patients and 192 downregulated (20 downregulated linear RNAs with *p*-adj < 0.05) (Figure 5B).

When the two different MS types are compared, similarly to what was observed for the circular transcriptome, remarkable differences were found. More than half of the linear transcripts comprise MS-type-specific profiles: 5067 linear RNAs are unique to RR-MS patients (39.2%) and 1756 linear transcripts (13.6%) can only be found in EVs from SP-MS patients (Figure 5C). Moreover, among those linear RNAs that can be found which are unrelated to the MS type, almost 300 are differentially expressed, with 204 upregulated (34 upregulated linear RNAs with *p*-adj < 0.05) and 92 downregulated linear transcripts in SP-MS patients (10 downregulated linear RNAs with *p*-adj < 0.05) (Figure 5C). Gene ontology analysis of DE linear transcripts in EVs has been performed without finding significant results.

### 3.6. miRNA Sponging Is Not the Primary Function of circRNAs in EVs and Leukocytes

The miRNA-sponging function is one of the most studied circRNA functions. However, it has been proposed that, to efficiently inhibit the function of a specific miRNA, a high number of binding sites (BS) for the target miRNA should be present in each circRNA molecule [20,21]. In order to assess whether the circRNAs contained in EVs could potentially have the function of sequestering miRNAs, we have compared them with a set of 3051 circRNAs predicted to be “super sponges” by Dudekula et al., based on the criteria of having at least 20 binding sites for a single miRNA [49].

**Figure 6:**
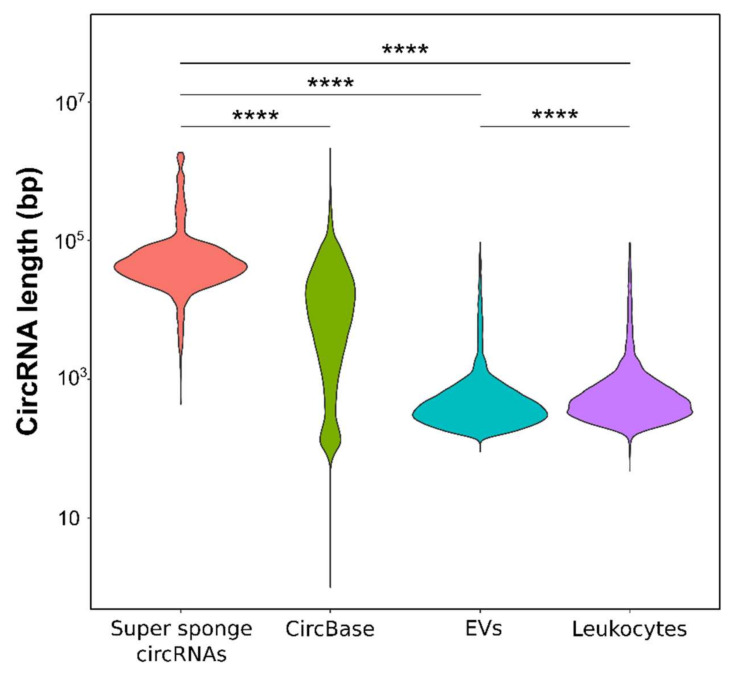
CircRNA length distribution comparison. The “super sponge” circRNA dataset retrieved from CircInteractome is compared to circRNAs included in circBase and circRNAs detected in our EV and leukocyte samples. Significant differences with respect to the set of circRNAs retrieved from CircInteractome, as assessed by t-test, are indicated. Length differences between circRNAs in EVs and leukocytes are also depicted. **** *p* < 0.00001.

Interestingly, we observed that the “super sponge” circRNAs retrieved from CircInteractome, had a remarkably longer median length (more than 100 times longer) than the circRNAs in our EVs samples (43,826 bps vs. 386 bps) (*p* < 0.0001). In order to understand whether circRNAs in EVs are unusually short, we included circRNAs detected in the leukocyte dataset previously published by our group [38] and the general circRNA population described in circBase as references. CircBase data confirm that there is a wide distribution of circRNA lengths, and the median length (8683 bps) is significantly shorter (five times shorter) than that observed for super-sponge circRNAs (*p* < 0.0001). Moreover, circRNAs detected in leukocytes are longer than those found in EVs (476 bps vs. 386 bps, *p* < 0.0001) but still much shorter than the “super sponge” circRNAs and the overall circRNAs included in circBase (*p* < 0.0001) (Figure 6).

**Figure 7:**
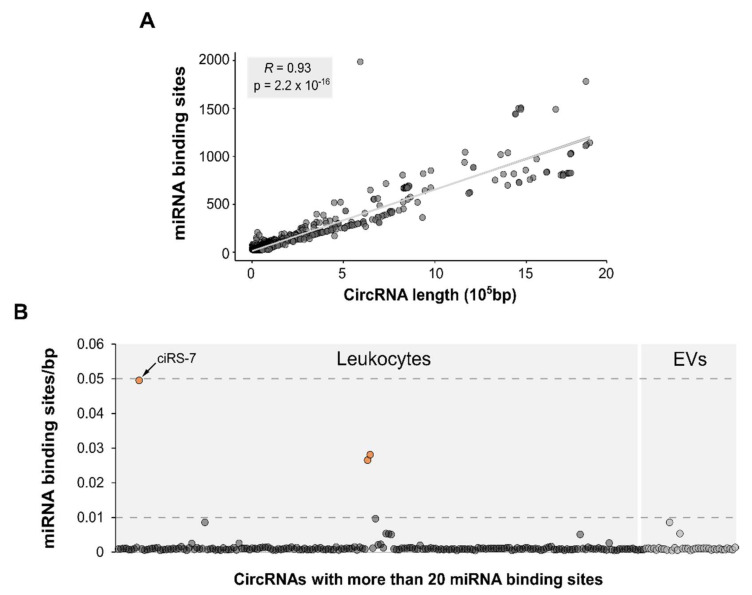
The number of miRNA binding sites (BS) generally increases with the length of the circRNA. (**A**) Pearson correlation analysis between circRNA length and the maximum number of BS for a single miRNA in the dataset retrieved from CircInteractome. (**B**) Ratio between the maximum number of miRNA BS for a single miRNA and the circRNA length for the circRNAs with more than 20 miRNA BS detected in leukocytes and EVs. CircRNAs with a ratio higher than 0.01 but smaller than 0.05 are highlighted in orange.

In light of this observation, we assessed whether there is a correlation between the circRNA length and the number of miRNA BS among the 3051 circRNAs retrieved from CircInteractome. Pearson correlation analysis confirmed a strong linear relationship (R = 0.93 and *p* = 2.2 × 10^−16^), indicating that the number of miRNA BS proportionally increases with the length of the circRNA (Figure 7A). Despite the short length of the circRNAs identified in our samples, a few circRNAs, 36 in EVs and 200 circRNAs in leukocytes, were found to be potential super sponges, based on Dudekula and colleagues’ definition [49] (more than 20 miRNA BS). However, this is only a theoretical approach, and even if a number of circRNAs are predicted to have more than 20 BS, they may have been accumulated by chance, and the sponging function of these circRNAs must be experimentally demonstrated. So far, the sponging function of very few circRNAs has been consistently proven. For instance, the miRNA-sponging function of CiRS-7 has been extensively demonstrated [55], and it contains a total of 70 BS for miR-7 within its 1485 bp, which could be taken as example of circRNA with more BS than expected by chance. Taking CiRS-7 as a reference, and knowing that its miRNA binding-site density is 0.05 (70 binding sites/1485 bp), we seek circRNAs that could have a similar miRNA binding-site density among the circRNAs detected in EVs, as a refined criteria for “super sponge” candidates. None of the circRNAs detected in EVs show a binding-site density of 0.05, nor even of 0.01 (1 binding site/100 bp). These results are in line with what we also find in the leukocyte circRNA dataset, where only three circRNAs have a miRNA BS density higher than 0.01, one of them being CiRS-7 (Figure 7B).

### 3.7. Highly Structured circRNAs Are More Frequent in Leukocytes than in EVs

Recent publications have drawn attention to circRNA structure as a determinant feature that can define their interaction with different proteins, and thus, their function [28,53]. In this line, Liu et al. reported that circRNAs containing double-stranded RNA (dsRNA), which will be called “highly structured circRNAs” from now on, are able to bind the dsRNA-activated protein kinase (PKR) and maintain it in an inactive state so that the activation of the innate immune response is prevented. Moreover, this PKR–circRNA interaction was proposed to regulate the inappropriate autoimmune reactions in patients with lupus. Therefore, with the aim of interrogating whether highly structured circRNAs could be enriched in EVs, we predicted the structure of the circRNAs detected both in EVs and leukocytes based on the length-normalized minimum thermodynamic free energy (−∆G/nt) [53].

**Figure 8:**
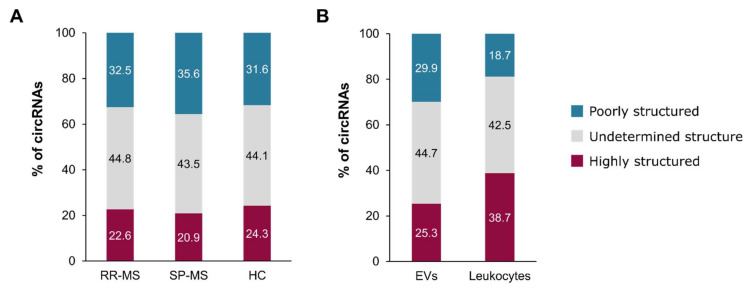
Structure prediction for circRNAs based on their −∆G/nt. Three different structure categories are defined: undetermined structure, highly structured and poorly structured. (**A**) Structure distribution of the circRNAs detected in EVs from RR-MS, SP-MS or HC individuals. Significant difference is found between SP-MS patients and HCs (*p* = 0.0036). (**B**) Comparison of the structure distribution between EVs and leukocytes, showing a significant difference between them (*p* < 0.001).

We observed that in EVs the majority of circRNAs have a −∆G/nt, based on which, following the criteria described by Fisher et al. [53], their structure cannot be reliably determined. Among the remaining circRNAs, the poorly structured ones represent about 31.6–35.6% of circRNAs, which are slightly more prevalent than the highly structured ones, ranging from 20.9 to 24.3%. Interestingly, this proportion does not differ much with the disease status, but still a significant difference is reported for the distribution between SP-MS patients and HCs (*p* = 0.0036) (Figure 8A).

Additionally, the comparison of the circRNA structure distribution between EVs and leukocytes shows that highly structured circRNAs tend to be preferentially retained in the cell (38.7% HS circRNAs in leukocytes vs. 25.3% in EVs), while poorly structured circRNAs are more frequent in EVs (18.7% PS circRNAs in leukocytes vs. 29.9% in EVs) (*p* < 0.0001) (Figure 8B).

## 4. Discussion

The EV transcriptome profile in MS was characterized back in 2017 by Selmaj et al., where they used RNASeq to study the expression of several classes of transcripts including small RNAs, and with a particular interest in miRNAs. This study revealed that exosomes, or small EVs, have a distinct RNA profile in RR-MS patients, and suggested that miRNAs might be a biomarkers for relapses [12]. Nevertheless, they focused their work on linear RNAs and did not study the circular transcriptome.

In this study, we present a genome-wide characterization of the transcriptome in EVs, including for the first time the circular transcriptome. We did not create a particular library preparation to specifically sequence small RNAs, but we still could detect some pre-miRNAs and snoRNAs (mainly ranging from 60 to 120 bps and from 80 to 200 bps, respectively). Despite the technical differences between studies, we confirmed that protein-coding genes comprise the most prevalent type of transcripts in EVs, while pre-miRNAs are quite scarce (0.01% of all the transcript types). In contrast, we found that circRNAs are abundant in EVs, which is in line with what has been found in other studies [18,19]. Indeed, circRNAs represent the second most abundant transcript in EV samples from MS patients and healthy controls. Moreover, it is important to mention that the circRNA abundance is probably underestimated, due to the fact that only reads spanning the BSJ are unique to circRNAs, and thus, only those are accounted as circRNA mapping reads. So, many reads mapping to the remaining sequence of the circRNA are assigned to other linear transcripts derived from the same gene. These transcripts are often protein-coding transcripts with sequences in common with the circRNA, potentially also leading to an overestimation of this type of transcript.

Previous studies on EV RNA content have reported that cell transcriptomes are only partially reflected in EV RNA cargo [56,57], which sometimes differs substantially from the RNA profile of the cell of origin [58,59,60]. Additionally, and regarding circRNAs, Li et al. have also reported that circRNAs were enriched in EVs compared to the secreting cells in cell culture [18]. In this context, and taking advantage of the transcriptome profile already performed in leukocytes by our group following the same analysis pipeline [38], we have compared the RNA profile in leukocytes and in plasma-derived EVs. When making these comparisons for plasma-derived EVs, it is important to take into account that EVs from different cell types enter the bloodstream, and thus, plasma EVs comprise a complex mixture of EVs from different origins. Nevertheless, a recently published study analysing RNA-Seq results from 101 plasma samples concluded that only 0.2% of plasma EVs were derived from other tissues, with 99.8% of circulating EVs generated from hematopoietic cells, and 45% particularly from leukocytes [61]. Thus, we can assume that a big proportion of the EVs analysed in the present study are from leukocyte origin, and we will discuss the differences between EV and leukocyte transcriptomic profiles as if they were the main secreting cells, although conclusions drawn from this comparison should be taken with caution.

In our study, we have found differences in the RNA biotypes between leukocytes and EVs. Remarkably, circRNAs are overrepresented in EVs (4.2% of the total reads, 58.4% of the reads corresponding to ncRNAs) compared to leukocytes (0.2% of the total reads and 0.9% of the ncRNA reads) (Figure 3). In order to understand the reasons driving the preferential incorporation of circRNAs into EVs, different factors have to be taken into account. In fact, cell abundance, specific sequence motifs, secondary structure, length, differential affinity for membrane lipids or association with RNA-binding proteins (reviewed in [60]) are some of the factors that have been reported to determine the RNA packaging into EVs.

Among these factors, high abundance is thought to favour the incorporation of a given RNA type into EVs [60]. In our data, this premise is true for protein-coding transcripts, which are the most abundant transcript in cells and also in EVs, but not for circRNAs, whose abundance is very low in cells but are still preferentially loaded into EVs. Therefore, some other factor must be favouring the circRNA enrichment in EVs. In terms of length, the range of circRNAs detected in cells and EVs is similar (100 to 90,000 bps), indicating that there is no physical limit for packaging large circRNAs into EVs. Nevertheless, it is worth noting that most of the bona fide circRNAs are between 250 and 800 bps, and longer circRNAs are exceptional. Interestingly, even if there is no apparent limit, a significant bias towards incorporating smaller circRNAs in EVs is observed (386 bps median length in Evs vs. 475 bps in leukocytes) (Figure 6). A similar length bias had previously been reported by Preuβer et al., who stated that length could be an important determinant for selective vesicle export of circRNAs, considering that the available packaging volume within a EV might be limiting to accommodate not only the circRNAs, but also the proteins associated with them [62]. Apart from the length, their circular structure may also help their condensation and favour their loading into EVs. However, based on our observations, the presence of double-stranded regions and hairpins seems to hinder their incorporation, resulting in a higher proportion of highly structured circRNAs in cells (Figure 8).

Interestingly, highly structured circRNAs have been described to be able to regulate the innate immune response in Systemic Lupus Erythematosus patients by binding and inhibiting the activation of the PKR [28]. Therefore, it is tempting to think that although they could be packaged into EVs more easily, highly structured circRNAs are preferentially accumulated in leukocytes in order to regulate the aberrant activation of the immune system, particularly in MS patients, where they have been found to be upregulated [38]. Similarly, in case circRNAs could be functional miRNA sponges, this functionality could influence their release into EVs. It is now widely accepted that a high number of binding miRNA sites are needed in order to exert the sponge function [20,21]. In this work, we have performed a correlation analysis, showing that most of the circRNAs predicted to have a high number of binding sites are unusually long, indicating that they may be accumulated by chance (Figure 7). Moreover, most of the bona fide circRNAs detected in our EV and leukocyte datasets, as well as circRNAs validated by others, are a few hundred nucleotides long (Figure 6) [63,64], indicating that long circRNAs (>1 kb) should be viewed critically, as they may be false positives. Taking these observations into account, and in light of the features of the best characterized and validated miRNA sponges, we suggest the miRNA BS density (number of binding sites/length) as a more accurate parameter for predicting the miRNA-sponge potential of circRNAs. Based on the density of miRNA binding sites, and in line with what has been suggested, only a very limited number of circRNAs have the potential to sponge miRNAs [20,21]. After calculating the miRNA BS density, the miRNA-sponging potential appears to be negligible among the circRNAs in leukocytes and in EVs, thus it is unlikely to influence the circRNA loading into EVs.

Besides intrinsic features of circRNAs which could favour or hinder their incorporation into EVs, the state of cells also impacts EV RNA profiles, and a given RNA could be preferentially packaged or not due to its physiological or pathological implications. In this line, beyond characterizing the general transcriptome profile of EVs in comparison to that from leukocytes, we have also characterized the differences between EVs from healthy donors and MS patients. In agreement with what Selmaj et al. previously reported [12], we have also found differences in the RNA cargo of EVs between MS patients and healthy controls, as well as between RR-MS and SP-MS patients. It is interesting to highlight that we have previously reported a global upregulation of circRNAs in MS patients’ leukocytes when compared to healthy controls [38]. Therefore, a global upregulation of circRNAs could also have been expected in EVs from MS patients, but in this case, we did not find a clear deregulation trend (Figure 4). This result could suggest that the upregulation of circRNAs may be playing an important role in the pathological context of MS leukocytes (such as their interaction with PKR), but not in EVs.

Anyway, both circular and linear transcripts differentially expressed between MS patients and healthy controls as well as transcripts that are unique to the diseased conditions could potentially be used as minimally invasive biomarkers of the disease. In fact, it is worth noting that one of the circRNAs found to be altered in RR-MS patients’ EVs when compared to controls’ EVs (circNEIL3) has previously been suggested as a biomarker candidate in leukocytes from RR-MS patients [38]. In contrast, in leukocytes, a very small proportion of the circRNA profile was different between RR-MS and SP-MS patients, and consequently, no potential biomarkers of conversion could be found [38]. Regarding the EV transcriptomic profiles characterized in the present study, several transcripts could distinguish between RR-MS and SP-MS patients (Figure 4 and Figure 6). The lack of significant results in the GO analysis could be due to the fact that GO analysis foundations may not be applicable to EVs. Nevertheless, all the results and candidates described in this work should be validated within a bigger cohort.

## 5. Conclusions

To the best of our knowledge, this study is the first to report the presence of 6575 circRNAs in plasma-derived EVs from MS and HC individuals. Based on the results drawn from the comparison of the transcriptomic profile between EVs and leukocytes, we suggest that the loading of circRNAs into EVs is a regulated process. Thus, the selective release of circRNAs into EVs could be implicated in the regulation of the physiopathology of the disease, although further research is needed.

Moreover, we report a number of linear and circular RNAs that are differentially expressed between MS and healthy controls or between RR-MS and SP-MS patients, which should be further studied in order to evaluate their potential role as biomarkers of MS.

## Figures and Tables

**Table 1:** Main clinical and demographical characteristics of individuals enrolled in the study classified by disease status.

Disease Status	Sex	Age	EDSS	Evol. Time	AOO
RR-MS (*n* = 10)	Male (*n* = 5)	39.7 (±13.7)	2 (0–3.5)	16.1 (±9.8)	23.6 (±9.1)
	Female (*n* = 5)	42.0 (±19.8)	0 (0–2)	13.2 (±12.2)	28.8 (±9.9)
SP-MS (*n* = 10)	Male (*n* = 5)	54.8 (±6.2)	6 (6–8.5)	20.7 (±8.7)	34.1 (±9.5)
	Female (*n* = 5)	48.7 (±6.7)	7 (4–8)	22.5 (±5.0)	26.2 (±6.6)
HC (*n* = 8)	Male (*n* = 4)	56.1 (±11.9)	-	-	-
	Female (*n* = 4)	53.6 (±11.8)	-	-	-

Abbreviations: RR-MS, relapsing–remitting multiple sclerosis; SP-MS. Secondary–progressive multiple sclerosis; HC, healthy control; EDSS, Expanded Disability Status Scale; Evol. Time: Evolution time; AOO, Age of Onset. Age, evolution time and AOO data are presented as “average (standard deviation)”, EDSS data are shown as “median (range)”.

**Table 2:** Potential circRNA candidates to differentiate between RR-Ms and HC and between the two subtypes of MS. Id, log_2_-fold change value, *p*-adjusted value, if the transcript is expressed in both or in just one of the groups, position and gene symbol are shown.

**CircRNA Candidates RRMS vs. HC**				
**Position**	**Gene Symbol**	**Expr. in**	**log_2_ FC**	***p*-adj**
chr10:76153898-76158337	ADK	both	12.31	0.00242
chr20:36361280-36365886	CTNNBL1	both	11.34	0.00512
chr5:80911291-80948145	SSBP2	both	11.77	0.00906
chr2:24777257-24807429	NCOA1	both	11.70	0.00934
chr19:50902107-50902741	POLD1	both	−10.63	0.01198
chr3:129579782-129599402	TMCC1	HC	−24.32	NA
chr8:98731276-98735263	MTDH	HC	−29.98	NA
chr1:151611363-151611595	SNX27	HC	−24.66	NA
chr14:69588933-69616220	DCAF5	RRMS	22.08	NA
chr1:51869090-51874004	EPS15	RRMS	9.51	NA
chr9:115013208-115015068	PTBP3	RRMS	24.97	NA
chr10:25226112-25231365	PRTFDC1	RRMS	10.40	NA
chr2:219394678-219411021	USP37	RRMS	10.58	NA
**CircRNA Candidates RRMS vs. SPMS**				
**Position**	**Gene Symbol**	**Expr. in**	**log_2_ FC**	***p*-adj**
chr10:76153898-76158337	ADK	Both	12.34	0.00172
chr15:89856134-89857938	FANCI	Both	−10.64	0.00683
chr20:36361280-36365886	CTNNBL1	Both	10.75	0.00794
chr7:77200394-77221573	PTPN12	Both	10.26	0.00818
chr18:76886266-76914555	ATP9B	Both	−9.95	0.01702
chr1:1735857-1756938	GNB1	RRMS	10.07	NA
chr16:53288349-53308214	CHD9	RRMS	9.69	NA
chr18:18586311-18588155	ROCK1	RRMS	9.19	NA
chr19:30476129-30477324	URI1|C19orf2	RRMS	9.17	NA
chr3:125216184-125223588	SNX4	RRMS	8.59	NA
chr18:67534592-67535352	CD226	SPMS	−37.13	NA
chr4:56265252-56269505	TMEM165	SPMS	−23.24	NA

## Data Availability

The datasets supporting the conclusions of this article will be available in the GEO repository for the time of publication or under request of the editor for peer review.

## Supplementary Materials

The following are available online at https://www.mdpi.com/article/10.3390/biomedicines9121850/s1, Figure S1: Diagram showing the workflow followed for the RNA-Seq from sample preparation to differential expression.
